# Pancreatic cancer-derived exosomes promoted pancreatic stellate cells recruitment by pancreatic cancer

**DOI:** 10.7150/jca.27590

**Published:** 2019-07-23

**Authors:** Yue-Feng Zhang, Yi-Zhao Zhou, Bo Zhang, Shi-Fei Huang, Peng-Ping Li, Xiao-Man He, Guo-Dong Cao, Mu-Xing Kang, Xin Dong, Yu-Lian Wu

**Affiliations:** 1 Department of Surgery, Second Affiliated Hospital, Zhejiang University School of Medicine, Hangzhou, Zhejiang, P.R. China.; 2 Key Laboratory of Cancer Prevention and Intervention, China National Ministry of Education, Cancer Institute, Second Affiliated Hospital, Zhejiang University School of Medicine, Hangzhou, Zhejiang, P.R. China.; 3 Department of Surgery, Traditional Chinese Medical Hospital of Hangzhou, Hangzhou, Zhejiang, P.R. China.; 4 Department of General Surgery, Fourth Affiliated Hospital, Zhejiang University School of Medicine, Yiwu, Zhejiang, P.R. China.; 5 Department of Hepatobiliary Surgery, Renmin Hospital of Wuhan University, Wuhan, Hubei, P.R. China.

**Keywords:** Pancreatic cancer, Pancreatic stellate cells (PSCs), Exosomes, Recruitment

## Abstract

Cancer-associated fibroblasts (CAFs), which are an important component of the tumor microenvironment, have been identified in the blood circulation of patients with cancer metastasis, and metastatic cancer cells can recruit circulating CAFs. However, primary carcinoma sites usually regulate the behavior of metastatic cancer cells through exosomes. Here, we hypothesized that cancer-derived exosomes could enhance CAF recruitment. Exosomes secreted by pancreatic cancer cells (PANC-1 and MIA PaCa-2) were isolated and characterized. The ability of pancreatic cancer to recruit pancreatic stellate cells (PSCs) was assessed with Transwell assays *in vitro* and bioluminescent imaging in a mouse model *in vivo*, and the underlying molecular mechanism was also investigated. The results showed that pancreatic cancer cell-derived exosomes (Exo-Pan and Exo-Mia) promoted the pancreatic cancer recruitment of PSCs. This effect was mediated partially by the transfer of the exosomal protein Lin28B to the recipient cells to activate the Lin28B/let-7/HMGA2/PDGFB signaling pathway. These results suggested that exosomes derived from local cancer could promote the formation of distant metastases through transferring the exosomal protein Lin28B to the metastatic cancer cells.

## Introduction

Metastasis is a hallmark of malignant tumors and the major cause of cancer-associated death 1, 28. Metastasis is an intricate event that includes multiple sequential steps, and many studies have attempted to elucidate the complicated mechanisms of metastasis 1, 2. Cancer-associated fibroblasts (CAFs) are important components of the tumor microenvironment. It has been increasingly recognized that CAFs could affect some cancer cell features; for example, they can promote epithelial-mesenchymal transition (EMT), proliferation, migration, invasion, metabolic reprogramming and chemoresistance 3, 4, 5, 26. Recently, Zheng Ao et al captured and identified CAFs in the circulating blood from patients with metastatic breast cancer 6. Another study revealed that colorectal cancer cells could recruit circulating CAFs, and high SMC1A expression levels in colorectal cancer cells promoted this recruitment 7. Additionally, a study found activated pancreatic stellate cells (PSCs) derived from primary pancreatic cancer in metastatic sites in mouse models, and verified that cancer cells could induce PSCs to migrate through blood vessels by secreting platelet-derived growth factor (PDGF) 11. This study indicated that metastatic cancer cells have the ability to recruit circulating CAFs, which is associated with their capacity to form new colonies.

Exosomes are membrane-enclosed nanovesicles that transport diverse bioactive molecules, such as proteins, lipids, and microRNAs (miRNAs), from donor cells to recipient cells; thus, exosomes can modify the physiological state of the recipient cells 8, 9. Numerous studies have indicated that exosomes secreted by the primary sites of carcinoma could be internalized by metastatic cancer cells or other cells; then, these exosomes can mediate multiple systemic pathophysiological processes, including promoting survival, immunosuppression, and pre-metastatic niche development 9, 10.

Inspired by the previous results, we performed this study to investigate whether cancer-derived exosomes could enhance CAF recruitment and to explore the molecular mechanisms. This information will be helpful for understanding the details of the biologic behaviors of cancers. We chose immortalized PSCs and the pancreatic cancer cell lines PANC-1 and MIA PaCa-2 for our experiments. Our results show that the exosomes derived from PANC-1 and MIA PaCa-2 cells (Exo-Pan and Exo-Mia) promoted PSC recruitment through activating the Lin28B/let-7/HMGA2/PDGFB signaling pathway.

## Materials and Methods

### Cell culture

The pancreatic cancer cell lines BxPC-3, PANC-1, MIA PaCa-2, and AsPC-1 and the immortalized normal pancreatic duct cell line HPDE were purchased from the Cell Bank of the Chinese Academy of Sciences (Shanghai, China). PANC-1 and MIA PaCa-2 cells were cultured in DMEM (Gibco, USA), and BxPC-3, AsPC-1, HPDE cells were cultured in 1640 medium (Gibco, USA). Both DMEM and 1640 medium were supplemented with 10% FBS (Gibco, USA) and a 1× penicillin-streptomycin solution (Life Technologies, USA). A PSC (Human Pancreatic Stellate Cells, Cat. 3830) cell line was obtained from ScienceCell^TM^ and maintained in the recommended stellate cell medium. All cells were kept in a humidified incubator at 37°C with 5% CO_2_.

### Immunofluorescence analysis

Active PSCs were naturally obtained by culturing with the recommended stellate cell medium. Quiescent (non-activated state) PSCs were induced by stellate cell medium containing 10 µM All-trans Retinoic Acid (ATRA, Sigma, USA) for 7 days 32. Then, PSCs were seeded in 24-well plates (5,000 cells/well) and cultured overnight. The cells were fixed in 4% paraformaldehyde, permeabilized with 0.5% Triton X-100, and blocked with 5% BSA for 1 h at 37°C. Then, the samples were incubated with anti-α-SMA primary antibodies overnight at 4°C, washed 3 times with PBS, and incubated with a FITC-conjugated secondary antibody for another 2 h before being washed with PBS again. Subsequently, 20 µl DAPI (Sigma, USA) was added to stain the nuclei. Finally, PSCs were observed with a fluorescence microscope (Carl Zeiss, Germany).

### Exosome isolation

First, FBS was centrifuged at 11,000 g for 16 h to eliminate the exosomes; this supernatant was used as exosome-depleted FBS. Cells were maintained in complete medium until they reached 70-80% confluence. Then, the cells were transferred to medium with 10% exosome-free FBS. After 48 h, the supernatants were collected and centrifuged at 2,000 g for 10 min to remove the dead cells and debris and at 10,000 g for 30 min to remove the large extracellular vesicles (EVs). Next, the supernatants were concentrated with a 30KD ultrafiltration device (Millipore, Germany) and filtered through 0.22-µm filters (Millipore, Germany) to eliminate vesicles larger than 200 nm. The resulting supernatants were centrifuged at 110,000 g for 70 min in a Beckman Optima L-100 XP ultracentrifuge (Beckman Coulter, Germany). The exosome precipitates were washed with PBS and centrifuged for another 70 min. The concentrations of the exosomes were determined using a BCA protein assay kit (Thermo, USA). Finally, the exosomes were resuspended in PBS and stored at -80°C. The exosomes isolated from PANC-1 and MIA PaCa-2 cells were designated as Exo-Pan and Exo-Mia.

### Physical characteristics of the exosomes

The exosomes were stained with 2% negative uranyl acetate dye, and a Tecnai 12 Bio-Twin transmission electron microscope (Philips, Netherlands) was used to observe the morphology and size of the exosomes. The size distribution of the exosomes was measured by a dynamic light scattering (DLS) analysis with a Nano Zetasizer (Malvern, UK) according to the manufacturer's instructions. Exosome marker proteins (TSG101, CD63 and Alix) were examined by Western blotting.

### Exosome internalization

Exosomes were labeled with the green fluorescent probe PKH-67 (Sigma, USA) according to the manufacturer's instructions. PKH-67-labeled exosomes (30 µg/ml) were incubated with PANC-1 and MIA PaCa-2 cells (30,000 cells/well) in a 24-well plate. After 4 h, the cells were harvested and observed using the same method described for the immunofluorescence analysis.

### Transwell assay

Cell recruitment was determined using 24-well Transwell^®^ chambers (Costar, USA). Different pancreatic cancer cells and HPDE cells (1×10^5^ cells/well) were seeded in the lower chamber, and the lower chamber was filled with 500 µl 10% FBS supplemented medium. A total of 2×10^4^ PSCs were seeded in the upper chamber. After 48 h, the PSCs that had not passed through the filter were removed with cotton swabs. The lower surface of the filter was fixed in formaldehyde and stained with 2% crystal violet solution (Goodbio, China) to detect the PSCs that had migrated into the lower chamber. The conditioned medium (CM) used in this assay was obtained by collecting and centrifuging the supernatants of PANC-1 and MIA PaCa-2 cells and was designated as PANC-CM and MIA-CM, respectively. PSC recruitment was evaluated using the average number of migrated cells from 4 random fields.

### Western blot analysis

Cells and exosomes were lysed with RIPA lysis buffer (Sigma, USA) supplemented with a 1× protease inhibitor mixture (Thermo, USA). Protein concentrations were determined with BCA assays. The experimental procedures were performed according to standard protocols. Briefly, the proteins were separated on SDS-polyacrylamide gels and then blotted onto polyvinylidene difluoride (PVDF) membranes. The membranes were blocked with 5% milk for 1 h, incubated with specific primary antibodies overnight at 4°C and horseradish peroxidase (HRP)-conjugated secondary antibodies for 1 h at room temperature, and then subjected to chemiluminescence detection. Antibodies against PDGFR-β, Lin28B, HMGA2, TSG101, Alix and c-MYC were obtained from Abcam (USA). Antibodies against PDGFB were obtained from Sigma-Aldrich (Germany). Antibodies against β-actin, α-SMA and CD63 and secondary antibodies were obtained from Huabio (Hangzhou, China). Densitometry analyses of Western blots were performed with the Image-J software (NIH, USA), and the β-actin was used as the internal control.

### Quantitative real-time PCR (qRT-PCR)

Let-7a and let-7b expression levels were assessed by qRT-PCR. Total RNA was extracted with the TRIzol reagent (Invitrogen, USA). Concentration and purity were determined with a NanoDrop 1000 (Thermo Scientific, USA). cDNA was synthesized with RevertAid Premium Reverse Transcriptase (Thermo Scientific, USA), and qRT-PCR was performed with a Step One^TM^ System (Applied Biosystems, USA) using a Sensi Mix SYBR Kit (Bio-Rad, USA). Data analysis was performed with the 2^-ΔΔCt^ method. The small nuclear RNA (snRNA) U6 was selected as an endogenous control. The specific miRNA primers used are listed in the Table 1:

### Lentiviral construction and stable transfection

To establish stable Lin28B knockdown clones, negative control small hairpin RNA (shNC, 5'-TTCTCCGAACGTGTCACGT-3') and Lin28B shRNA (shLin28B, 5'-GCTACAACTGTGGTGGCCTTGATCA-3') were synthesized and cloned into lentivirus vectors to construct shNC and shLin28B lentivirus by Hanbio (Shanghai, China). For lentivirus transfection, PANC-1 cells were transduced with lentivirus at an infection MOI≥20. At 48 h after lentivirus infection, puromycin (2ug/ml) was used to select stable clones. PANC-1 cells that were transfected with shNC and shLin28B lentivirus were designated as PANC^NC^ and PANC^sh^, and the exosomes secreted by PANC^NC^ and PANC^sh^ cells were designated as Exo^NC^ and Exo^sh^, respectively.

### Animal experiments

The animal study was approved by the 2nd Affiliated Hospital of the School of Medicine of Zhejiang University Review Board. Four- to six-week-old BALB/c nude mice (Shanghai SLAC Laboratory Animals, China) were randomly divided into four groups (n=8) and maintained under specific pathogen-free (SPF) conditions. Liver metastatic mice models were induced by splenic injection 7. First, the mice were anesthetized by intraperitoneal chloral hydrate injection (0.4 mg/g). A 100-µl suspension of PANC-1 cells (5×10^5^ cells/mouse), Exo-Pan-treated PANC-1 cells (PANC-1/Exo-Pan, 5×10^5^ cells/mouse) or PBS was injected into the spleen of each mouse. After 24 h, a 100-µl suspension of dye-labelled PSCs (3×10^5^ cells/mouse) or PBS was injected through the caudal vein to simulate circulating CAFs. These groups were subjected to the following treatments: 1) Group a: PANC-1 cells alone, 2) Group b: PSCs alone, 3) Group c: PANC-1 cells and PSCs, 4) Group d: PANC-l cells/Exo-Pan and PSCs. After 48 h, the distributions of PSCs were assessed with an IVIS Lumina III *In Vivo* imaging system (Perkin Elmer, USA). Metastatic PANC-1 cells and PSCs in the liver were checked by immunofluorescence analysis. Five of 8 mice in each group were injected with wild-type PANC-1 cells and DiR-labelled PSCs and subjected to bioluminescence imaging. The remaining 3 mice in each group were injected with PANC^NC^ cells (expressing GFP) and CM-Dil-labelled PSCs, and liver slices were subjected to immunofluorescence analysis.

### Statistical analysis

Data were acquired from at least three independent experiments and are presented as the mean ± SD. Statistical Package for the Social Sciences version 21.0 (SPSS Inc., USA) was used for the statistical analyses. Unpaired *t* tests were used for the statistical analyses. Statistical significance was considered when *p<*0.05 (*p<0.05; **p<0.01; ***p<0.001). All histograms and curves were created with GraphPad Prism 6 (GraphPad Software, La Jolla, CA, USA).

## Results

### PSCs were in an activated state

**Figure 1:**
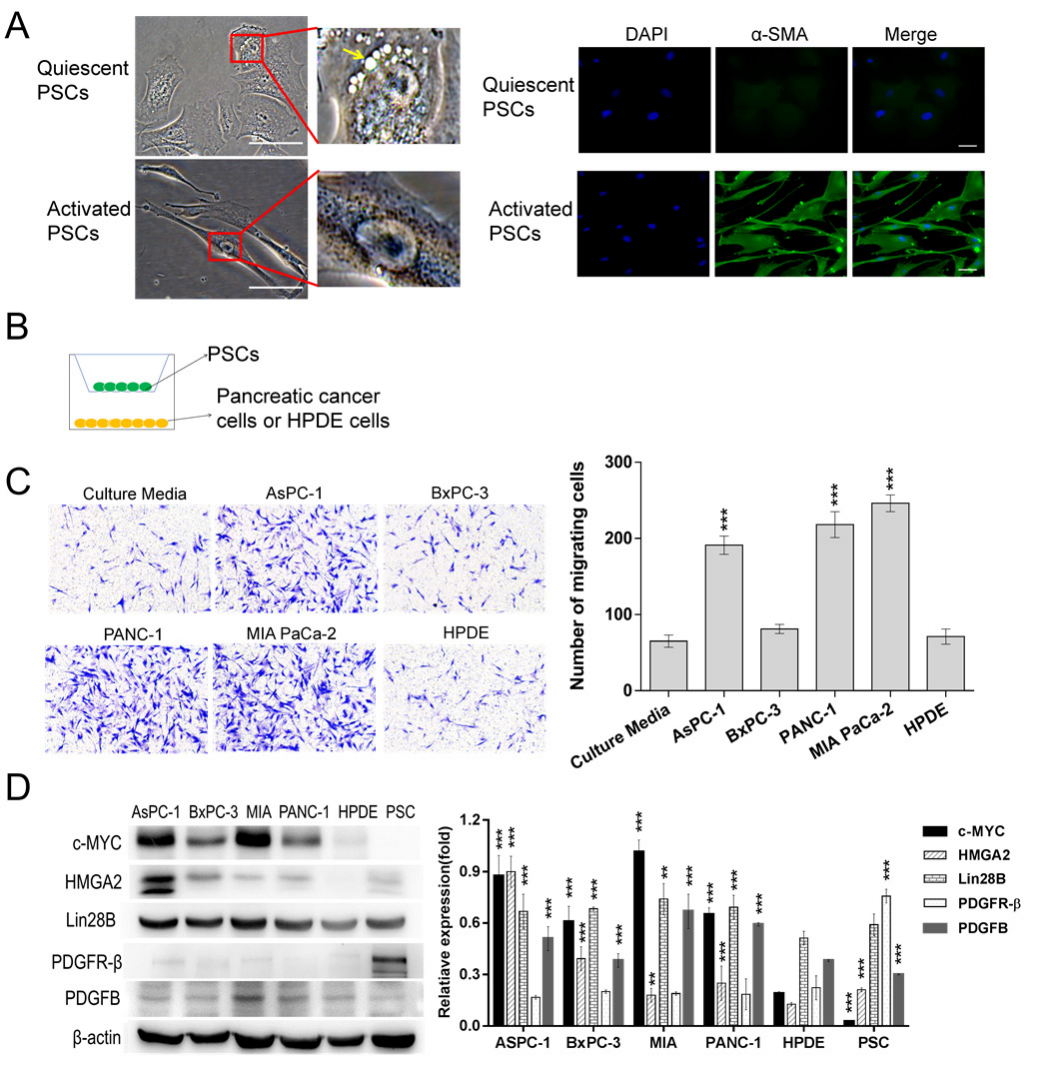
** Identification of activated PSCs and selection of pancreatic cell lines.** (A) Identification activated PSCs. Active PSCs were obtained by culturing with the recommended stellate cell medium. Quiescent PSCs were induced by ATRA. The left panel showed the typical morphology of PSCs, a yellow arrow indicates the lipid droplets in the ATRA-induced PSCs; the right panel presented the expression levels of α-smooth muscle actin (α-SMA) in PSCs (Scale bar, 100 µm). (B) Schematic diagram illustrating the Transwell assays. (C) The ability of different pancreatic duct cells to recruit PSCs was assessed with Transwell assays. PSCs cocultured with culture medium alone were used as a control. A total of 2×10^4^ PSCs were seeded in each upper chamber (***p<0.001). (D) Western blot analysis of c-MYC, Lin28B, HMGA2, PDGFR-β and PDGFB expression in different cell lines. The HPDE cells were used as a control (*p<0.05, **p<0.01, ***p<0.001).

PSCs are resident cells of the pancreas, and they remain quiescent (non-activated state) in a healthy pancreas 12, 13. In the context of chronic pancreatitis and pancreatic ductal adenocarcinoma (PDAC), PSCs are converted into an activated state and contribute to inflammatory and profibrogenic reactions 12, 13. As shown in Fig. 1A, ATRA-induced PSCs exhibited a polygonal or irregular shape with lipid droplets and expressed low levels α-smooth muscle actin (α-SMA), a cytoskeletal marker for activated PSCs, which matched the description of quiescent PSCs 12. In contrast, PSCs that were cultured with common stellate cell medium exhibited fibroblast-like characteristics with no vitamin A lipid droplets surrounding the central nucleus, and extensive green staining for α-SMA was observed in the cytoplasm. The results suggested that these PSCs were in an activated state.

### Pancreatic cells had different capacities for recruiting PSCs

The pancreatic cancer cell-induced recruitment effects on PSCs were assessed with Transwell assays, and a schematic diagram is shown in Fig. 1B. According to Fig. 1C, similar numbers of PSCs migrated to the lower chamber in the culture medium group and the BxPC-3 and HPDE groups. In contrast, significantly more PSCs passed through the filter in the AsPC-1, PANC-1 and MIA PaCa-2 groups. These data indicated that the AsPC-1, PANC-1 and MIA PaCa-2 cells efficiently recruited PSCs. Then, Western blots were conducted to detect c-MYC, Lin28B, HMGA2, PDGFB and PDGFR-β expression (Fig. 1D). PDGFR-β was highly expressed in PSCs and lowly or not expressed in the pancreatic ductal cell lines. PDGFB was detected in all of the cell lines. However, the AsPC-1, PANC-1 and MIA PaCa-2 cell lines had relatively higher PDGFB expression levels, which was in agreement with their stronger ability to induce PSC chemotaxis.

### Morphological characterization and identification of exosomes

**Figure 2:**
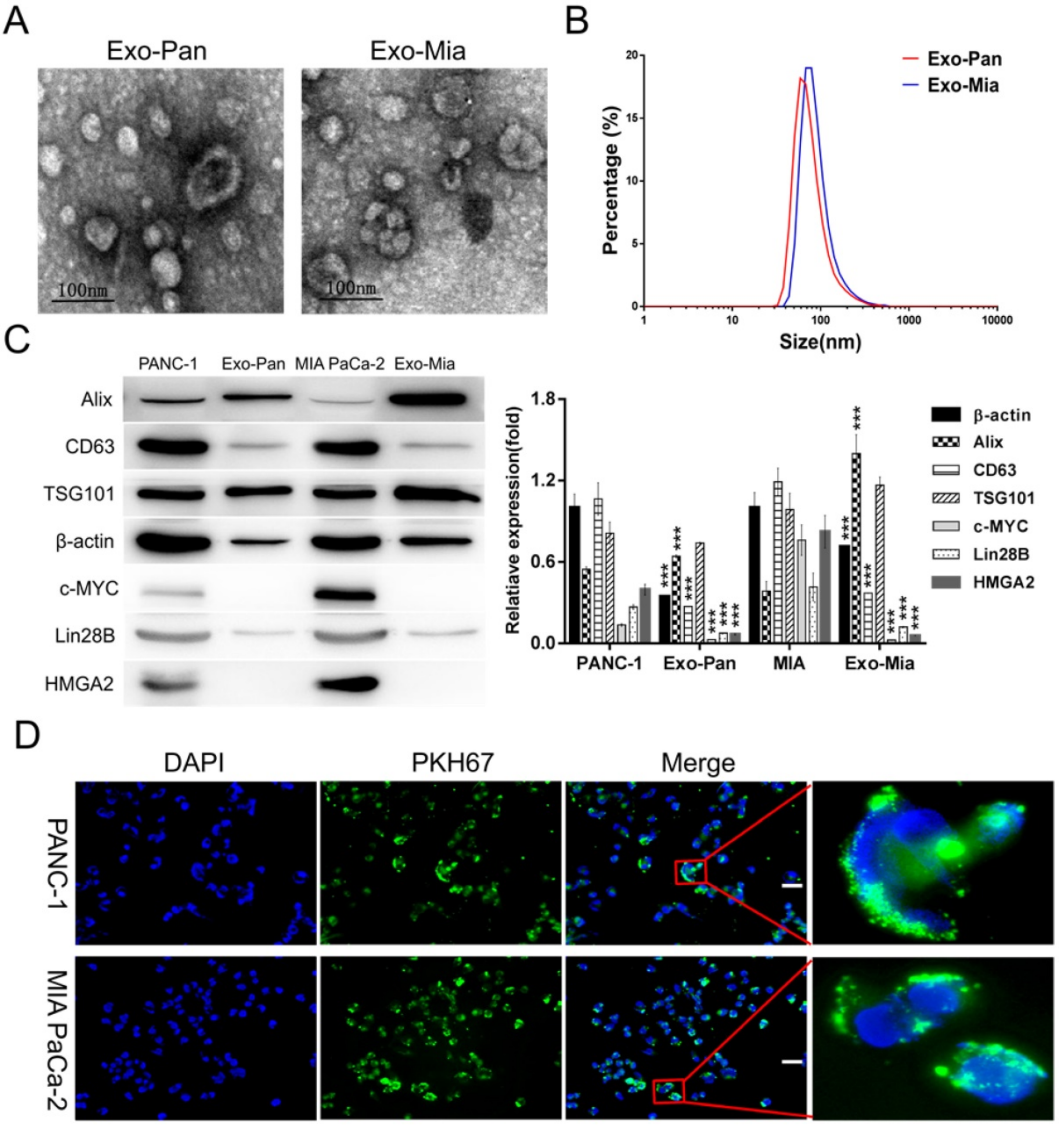
** Exosome characterization and exosome internalization by PANC-1 and MIA PaCa-2 cells.** (A) TEM images of Exo-Pan and Exo-Mia. (B) Size distributions of Exo-Pan and Exo-Mia. (C) Western blot analysis showing the presence of the exosomal markers Alix, TSG101, and CD63 and the levels of c-MYC, Lin28B, HMGA2, and β-actin. PANC-1 and MIA PaCa-2 cells were used as controls, respectively (***p<0.001). (D) Exo-Pan and Exo-Mia readily entered PANC-1 and MIA PaCa-2 cells. The cells were cocultured with the exosomes (30 µg/ml) for 4 h. (Scale bar, 100 µm).

Exosomes isolated from PANC-1 and MIA PaCa-2 cells were designated as Exo-Pan and Exo-Mia. Fig. 2A displays representative Exo-Pan and Exo-Mia transmission electron microscopy (TEM) images; most of the vesicles were spherical in shape and smaller than 100 nm. According to the Zetasizer measurements, the average diameter of the exosomes was approximately 60 nm (Fig. 2B). The Western blot results revealed that the exosome marker proteins (Alix, CD63 and TSG101) were enriched in both the cells and exosomes, and the nucleoproteins (c-MYC and HMGA2) were present in the cells but absent in the exosomes. Lin28B and β-actin were also present in both the cells and exosomes (Fig. 2C).

### Exo-Pan and Exo-Mia readily entered the PANC-1 and MIA PaCa-2 cells

The PANC-1 and MIA PaCa-2 cells were observed after exposure to 30 µg/ml PKH 67-dyed Exo-Pan and Exo-Mia for 4 h. As shown in Fig. 2D, the abundant green spots accumulated in the cytoplasm of these cells, which indicated that the PANC-1 and MIA PaCa-2 cells could efficiently uptake Exo-Pan and Exo-Mia.

### Exo-Pan and Exo-Mia promoted PSC recruitment by upregulating PDGFB

**Figure 3:**
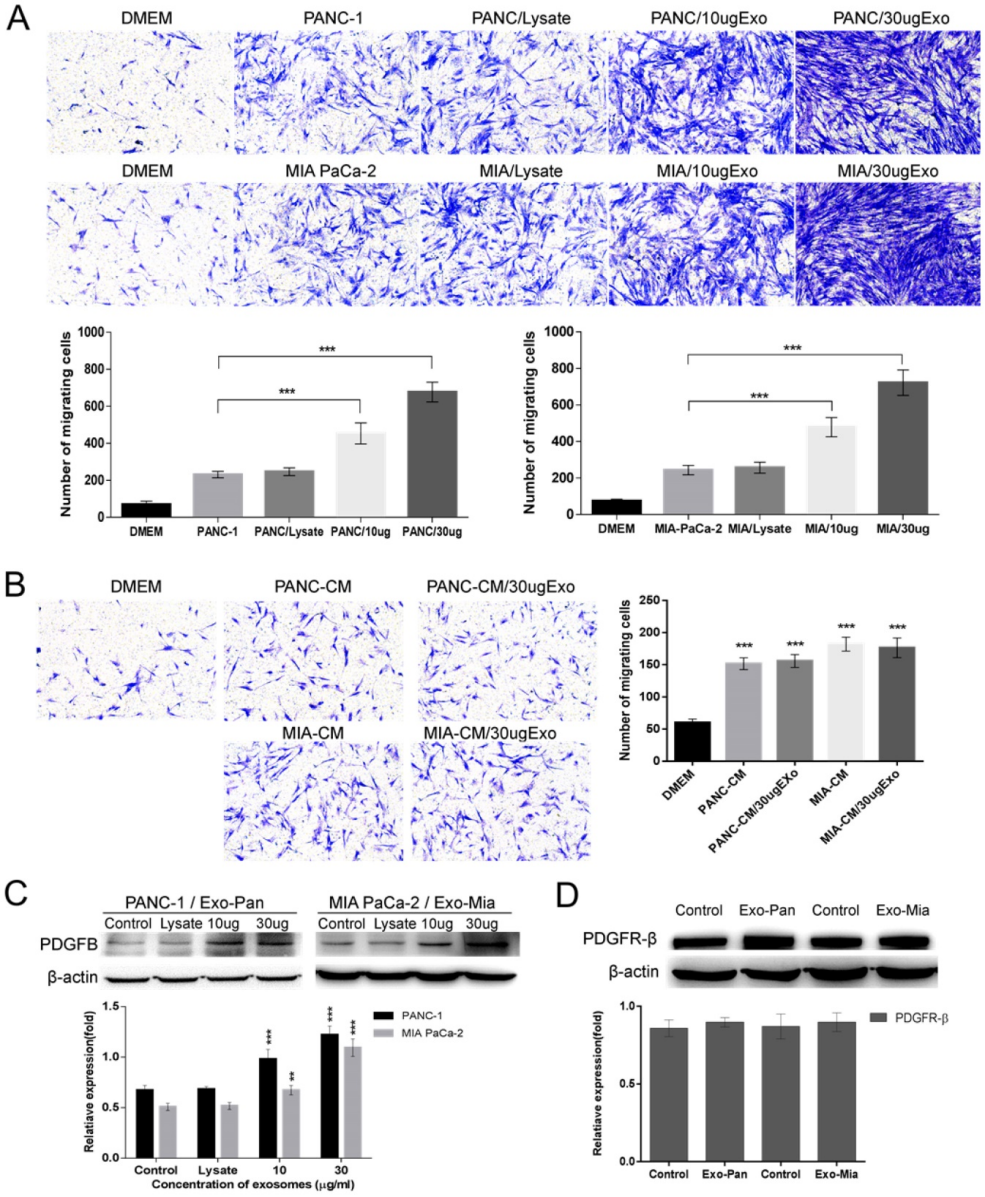
** Exo-Pan and Exo-Mia promoted PSC recruitment by upregulating PDGFB.** (A) PANC-1 and MIA PaCa-2 cells were treated with cell lysate or exosomes (Exo-Pan and Exo-Mia). Untreated PANC-1 and MIA PaCa-2 cells were used as control groups (***p<0.001). (B) The lower chambers were filled with conditional medium (CM) derived from PANC-1 cells (PANC-CM) and MIA PaCa-2 cells (MIA-CM), and cellular lysate or exosomes were added to the CM. The PSCs in the control group were exposed to fresh completed DMEM (***p<0.001). (C) Western blot analysis of PDGFB in the exosome-treated PANC-1 and MIA PaCa-2 cells. Untreated PANC-1 and MIA PaCa-2 cells were used as control groups (**p<0.01, ***p<0.001). (D) Western blot analysis of PDGFR-β expression levels in the PSCs treated with exosomes or not.

The effects of the exosomes on the ability of cancer cells to recruit PSCs were measured with Transwell assays. As shown in Fig. 3A, compared with the control cells, the co-culture of PSCs with PANC-1 or MIA PaCa-2 cells induced PSC migration to them. Upon adding exosomes to the PANC-1 or MIA PaCa-2 cells, markedly more PSCs passed through the filter and migrated to the lower chambers. The larger the dose of exosomes added, the more PSCs migrated. In contrast, no or a weakened enhancing effect was observed in the cellular lysis-treated group. To verify whether the exosomes alone could mediate PSC migration, PANC-1 and MIA PaCa-2 cells were replaced with their CM in the lower chambers. Interestingly, compared with the control conditions, the culture medium could induce weak PSC chemotactic migration, but the addition of exosomes caused no effects on PSC migration. Furthermore, the Western blot results demonstrated that exosomes treatment increased PDGFB expression in the pancreatic cancer cells in a concentration- dependent manner, but the exosomes had no effect on PDGFR-β expression in PSCs.

### Exo-Pan and Exo-Mia caused exosomal protein Lin28B transfer to the recipient cells

**Figure 4:**
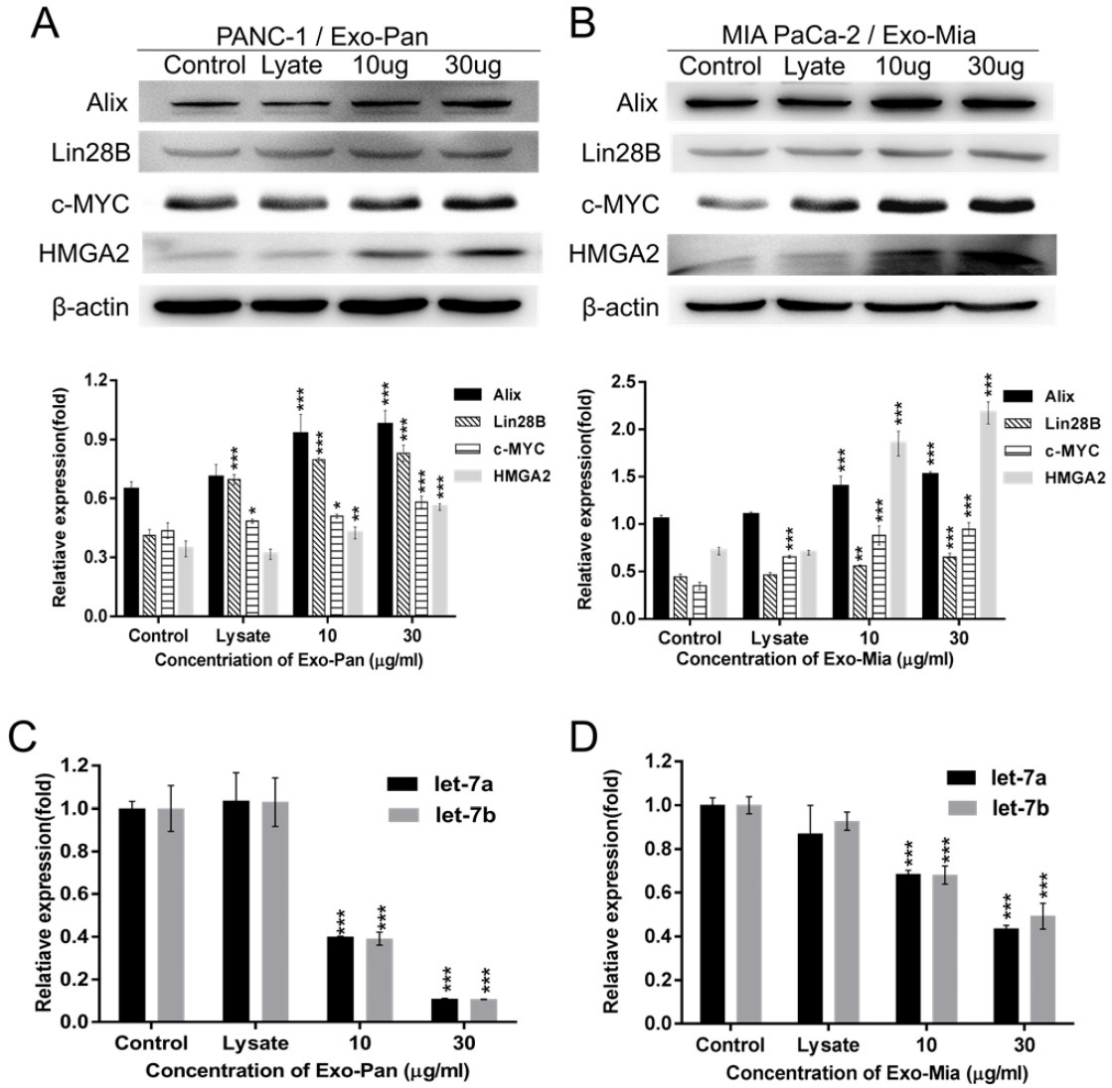
** Exosomes activated the Lin28B/let-7/HMGA2/PDGFB signaling pathway.** To verify Lin28B/let-7/HMGA2/PDGFB signaling pathway activation, we determined the expression of related molecules. PANC-1 (A) (C) and MIA PaCa-2 cells (B) (D) were treated with cellular lysate or exosomes. Untreated PANC-1 and MIA PaCa-2 cells were used as controls (*p<0.05, **p<0.01, ***p<0.001).

We hypothesized that the exosomal protein Lin28B played a role in promoting the PSC recruitment effect, so the expression of molecules involved in the Lin28B/let-7/HMGA2/PDGFB pathway was measured. Figs. 4A & B reveal that the expression levels of c-MYC, Lin28B, HMGA2 and PDGFB as well as the exosomal marker protein Alix were increased in the exosome-treated PANC-1 and MIA PaCa-2 cells in a dose-dependent manner. The results of qRT-PCR showed that exosome-treated PANC-1 and MIA PaCa-2 cells exhibited lower expression levels of let-7a and let-7b than the control group or lysis group (Fig. 4C & D). These results suggested that exosomes indeed activated the Lin28B/let-7/HMGA2/PDGFB signaling pathway* via* transferring exosomal Lin28B to the recipient cells.

### Exo^NC^ can restore the impaired PSC recruitment by PANC^sh^

**Figure 5:**
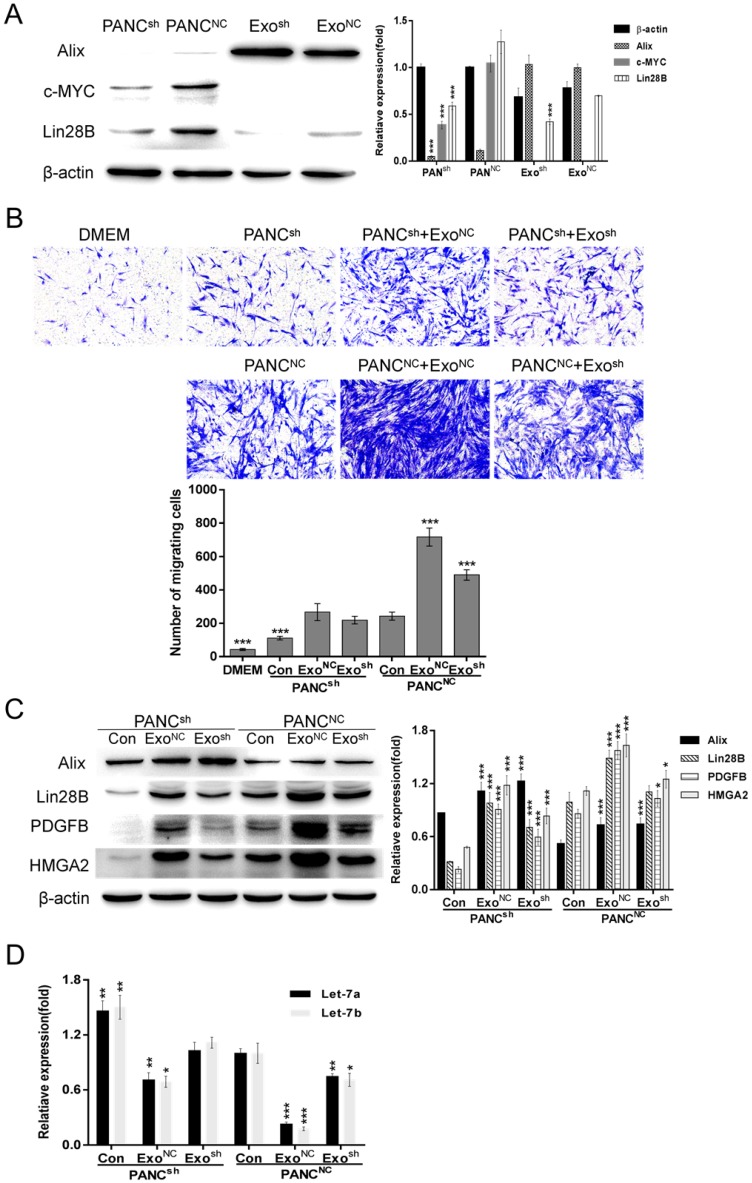
** Exo^NC^ could restore impaired PSC recruitment by PANC^sh^.** (A) Western blot analysis of Alix, β-actin, c-MYC and Lin28B expression in PANC^NC^, PANC^sh^, Exo^NC^ and Exo^sh^ cells. PANC^NC^ cells and Exo^NC^ were used as control, respectively (***p<0.001). (B) PANC^NC^ and PANC^sh^ cells were treated with 30 µg/ml Exo^NC^ and Exo^sh^, respectively. Their ability to recruit PSCs was assessed with Transwell assays; 2×10^4^ PSCs were seeded in each upper chamber. Untreated PANC^NC^ cells were used as the control group (***p<0.001). (C) and (D) Lin28B/let-7/HMGA2/PDGFB signaling pathway related molecules were identified. Untreated PANC^NC^ cells were used as control (*p<0.05, **p<0.01, ***p<0.001).

To further verify the key role that Lin28B plays in regulating PSC recruitment, we constructed stable Lin28B knockdown clones and a negative control: PANC^sh^ and PANC^NC^. As shown in Fig. 5A, the Lin28B expression levels were detected in both the cell types (PANC^sh^ and PANC^NC^) and their exosomes (Exo^sh^ and Exo^NC^), Lin28B expression was decreased in both the cells and exosomes compared to the negative control. After Lin28B knockdown, the expression of c-MYC was inhibited to some extent (Fig. 5A). Compared to PANC-1^NC^, attenuated PSC recruitment by PANC-1^sh^ was also be noticed in Fig. 5B. In addition, Exo-Pan^sh^ has the less ability than Exo^NC^ to promote the PANC^sh^ or PANC^NC^ to recruit PSCs. In contrast, Exo^NC^ could restore impaired PSC recruitment by PANC^sh^. The Lin28B/let-7/HMGA2/PDGFB signaling pathway analysis in Fig. 5C & D was consistent with the phenomenon in Fig. 5B.

### Exo-Pan enhanced PSC recruitment *in vivo*

**Figure 6:**
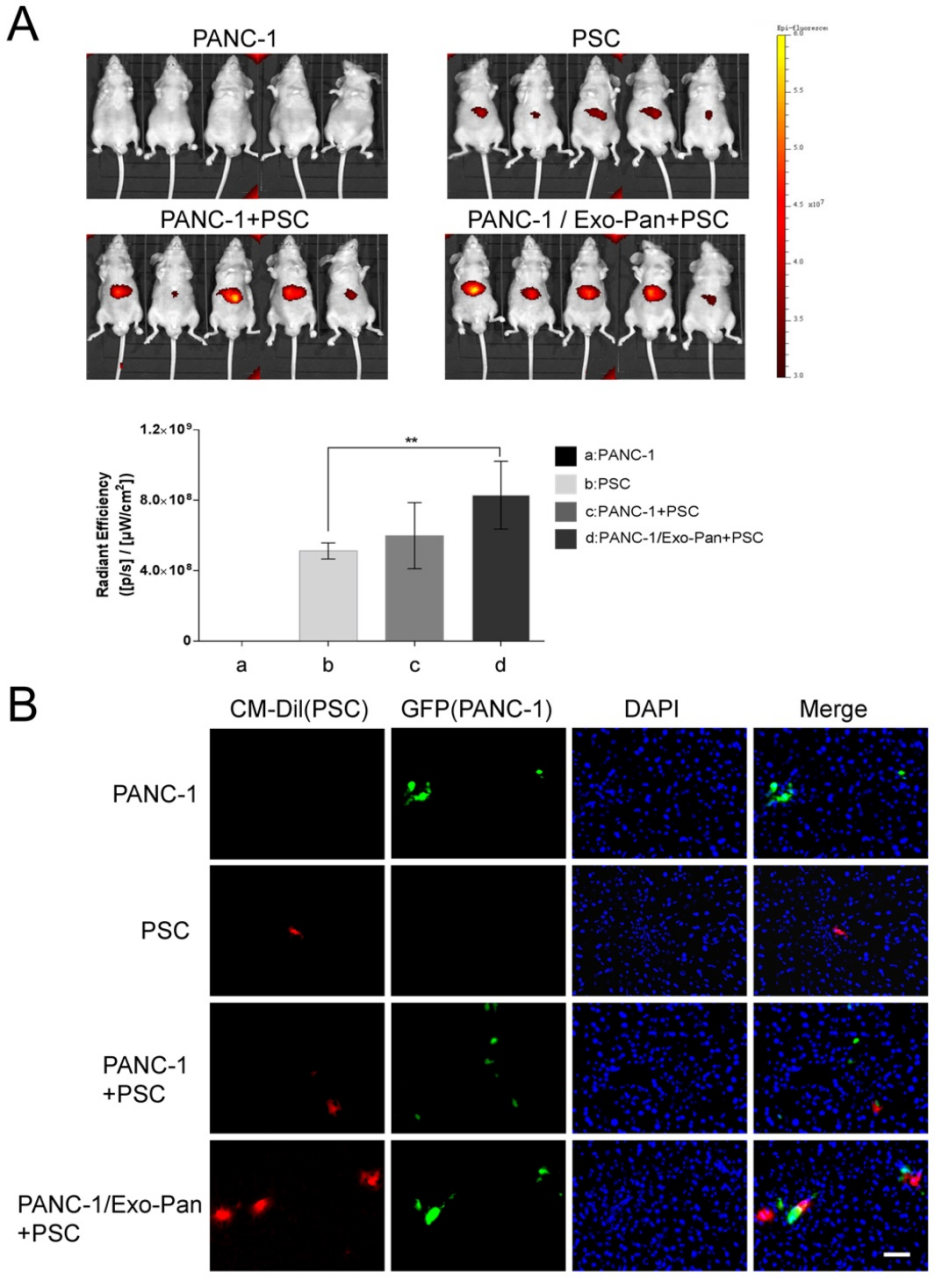
** Bioluminescent imaging of the mouse model *in vivo*.** (A) Representative bioluminescent images of the following groups: 1) Group a: PANC-1 cells alone, 2) Group b: PSCs alone, 3) Group c: PANC-1 cells and PSCs, and 4) Group d: PANC-l cells/Exo-Pan and PSCs. Red fluorescence indicates the distribution of PSCs (**p<0.01). (B) Immunofluorescence images of liver sections from different groups. Green fluorescence indicates PANC^NC^ cells that express GFP; red fluorescence indicates CM-Dil-labelled PSCs; and blue spots indicate nuclei stained with DAPI (Scale bar, 100µm).

The liver metastasis tumor model was established by injecting PANC-1 cells into the spleens of mice. Then, labeled PSCs were injected into the caudal veins to mimic circulating CAFs. The bioluminescent images reflect the location of PSCs. As shown in Fig. 6A, no fluorescence was detected in bodies of mice in Group a (PANC-1 cells alone), and weak fluorescence signals appeared in the upper abdomens of mice in Group b (PSCs alone). The fluorescence intensity was higher in Group c (PANC-1 cells and PSCs) than in Group b (PSCs alone), and the fluorescence signal was stronger in Group d (PANC-l cells/Exo-Pan and PSCs) than in the other groups (Fig. 6A). However, statistical significance for the fluorescence intensity results was obtained only between Group b (PSCs alone) and Group d (PANC-l cells/Exo-Pan and PSCs). The immunofluorescence images of liver slices further confirmed the presence of metastatic PANC-1 cells (green) and CM-Dil-labelled PSCs (red) in the liver. Relatively more PSCs were accumulated near the PANC^NC^ cells in Group d than in Group c (Fig. 6B). These results indicated that Exo-Pan enhanced the ability of PANC-1 cells to recruit circulating PSCs *in vivo*.

## Discussion

Cancer cells detach from primary tumors and enter the circulation during the early stage of the disease, but only a minority of these disseminated cells will survive and form metastatic colonies in distant organs 33. In addition to cancer cells, large amounts of exosomes and CAFs are simultaneously released into the circulation 6, 10. Increasing evidence has shown that interactions between cancer cells and these exosomes or CAFs alone will promote metastasis 5, 10, 26, 31, 34. We focused on the interactions among these three factors. The pancreatic cancer cell lines PANC-1 and MIA PaCa-2 and the corresponding cancer-associated fibroblasts lines of PSCs were used in the experiments. We verified that the pancreatic cancer cell-derived exosomes (Exo-Pan and Exo-Mia) could increase the expression level of PDGFB and promote pancreatic cancer recruitment of PSCs.

Lin28B is a homolog of Lin28, and both are highly conserved RNA-binding proteins 35. Lin28B overexpression is involved in tumorigenesis in various cancers, including hepatocellular carcinoma, esophageal cancer, colorectal cancer, and pancreatic cancer 18, 23, 24, 25. Lin28B is a well-known suppressive regulator of miR let-7, and the expression of miR let-7 is inversely correlated with HMGA2/PDGFB expression, which indicates that Lin28B could indirectly regulate PDGFB expression *via* the Lin28B/let-7/HMGA2/PDGFB signaling pathway 18, 19, 27. As shown in Fig. 2C, Lin28B was detected in both the cells and their exosomes (Exo-Pan and Exo-Mia). Therefore, it is likely that Exo-Pan and Exo-Mia affected the PANC-1 and MIA PaCa-2 cells by transferring the exosomal protein Lin28B to the recipient cells. As expected, the expression levels of Lin28B, HMGA2 and PDGFB, as well as the exosomal marker protein Alix, were increased in the exosome-treated PANC-1 and MIA PaCa-2 cells (Fig. 3D & Figs. 4A & B). Moreover, exosomes downregulated the expression of miR let-7a and let-7b in the exosome-treated group (Fig. 4C & D). Although c-MYC is a nucleoprotein, it did not appear to be an exosomal protein (Fig. 2C). The expression levels of c-MYC in exosome-treated PANC-1 and MIA PaCa-2 cells were obviously increased (Figs. 4A & B), which was attributed to the fact that c-MYC expression is also associated with the Lin28B/let-7 signaling pathway 18, 29. Furthermore, Exo^sh^ could not induce PANC^sh^ or PANC^NC^ to recruit PSCs as the Exo^NC^ did; however, Exo-Pan could restore impaired PSCs recruitment by PANC^sh^ (Fig. 5). These results confirmed that Exo-Pan and Exo-Mia indeed activated the Lin28B/let-7/HMGA2/PDGFB signaling pathway by transferring Lin28B.

In addition, we also found Exo-Pan or Exo-Mia alone could not facilitate PSC migration, and they only exert their effects by mediating PANC-1 and MIA PaCa-2 cells (Fig. 3B). In our opinion, the migration of PSCs to pancreatic cancer cells is a chemotactic process, and PDGFB is a well-studied and powerful chemokine in PSCs 14, 15, 16, 17, 30. PDGFB is secreted by pancreatic cancer cells and binds to the PDGFR on the surface of PSCs, enhancing their migration 16, 17. The concentration of PDGFB is increased with decreasing distance from pancreatic cancer cells, which consequently leads to more intensive migration of PSCs.

However, the exosomes are relatively stable extracellular vesicles, and no study has observed that exosomes can bind to and activate PDGFRs. In contrast, PDGFR-β is highly expressed in PSCs, and little or no expression occurs in pancreatic cell lines (Fig.1D). This suggests that pancreatic cancer-derived exosomes contain few or no downstream molecules of PDGFRs; therefore, even though the exosomes were internalized by PSCs, the PDGFB/PDGFR signaling pathway could not be activated. In summary, the exosomes were not chemokines for PSCs and could not affect their migration.

Metastasis is a common but complex phenomenon of malignant tumors. Many details and mechanisms remain to be elucidated 1, 28, 30. Increasing evidence, including the present study, shows that primary tumors can not only release cancer cells into the blood, but also “dispatch” exosomes and CAFs as their “accomplices” 6, 10, 20, 33. Complicated interactions occur among these factors that eventually facilitate the formation of new colonies. This new concept of metastasis will provide an alternative strategy for cancer therapy.

In conclusion, pancreatic cancer cell-derived exosomes could transfer the exosomal protein Lin28B to pancreatic cancer cells, thus facilitating their recruitment of PSCs *via* the Lin28B/let-7/HMGA2/PDGFB pathway. This study suggested that the exosomes derived from local cancer cells could promote the construction of distant metastases through transferring the exosomal protein Lin28B to metastatic cancer cells.

## Figures and Tables

**Table 1:** The specific miRNA primers are listed in the table.

Gene	Primer sequences
let-7a-F	5'-GCTGAGGTAGTAGGTTGTATAGTT-3'
let-7a-R	5'-GTGCAGGGTCCGAGGT-3'
let-7b-F	5'-GGGTGAGGTAGTAGGTTGTGTGGT-3'
let-7b-R	5'-GTGCAGGGTCCGAGGT-3'
U6-F	5'-TGGCACCCAGCACAATGAA-3'
U6-R	5'-CTAAGTCATAGTCCGCCTAGAAGCA-3'

## Abbreviations

PDAC: pancreatic ductal adenocarcinoma
PSCs: pancreatic stellate cells
CAFs: cancer-associated fibroblasts
EMT: epithelial-mesenchymal transition
α-SMA: α-smooth muscle actin
ATRA: all-trans retinoic acid
PDGFB: platelet-derived growth factor B
PDGFR-β: platelet-derived growth factor receptor-β
HMGA2 or HMGI-C: high mobility group AT-hook 2 or high mobility group protein isoform I-C
miRNA: microRNA
